# Society Meetings

**Published:** 1903-09

**Authors:** 


### SOCIETY MEETINGS.

The American Association of Obstetricians and Gynecologists will hold its sixteenth annual meeting in the Northwestern University Medical School Building, Chicago, Ill., Tuesday, Wednesday and Thursday, September 22, 23 and 24, 1903, under the presidency of Dr. Lehman H. Dunning, of Indianapolis.

The Auditorium Hotel, Annex, has been selected for the headquarters of the association, the management of which should be addressed concerning rooms and rates.

Dr. J. B. Murphy, Reliance Building, 100 State Street, is the chairman of the committee of arrangements and will gladly furnish any information to members and guests upon application. Dr. Murphy also can be addressed, relating to accommodations, at the Auditorium Annex, or other hotels.

### SCIENTIFIC PROGRAM.

The following list of papers has been offered:

1. President's address, L. H. Dunning, Indianapolis.
2. Supravaginal amputation for fibroids, with report of cases, H. E. Hayd, Buffalo.
3. Traumatic rupture of intestines without external marks of violence, with report of cases, Geo. S. Peck, Youngstown, O.

4. Ectopic pregnancy, H. D. Ingraham, Buffalo.

5. Relationship of the colon to abdominal tumors, J. F. Baldwin, Columbus.

6. Cysts of the kidney, resembling ovarian tumors, with cases, Rufus B. Hall, Cincinnati.

7. Total extirpation of the vagina for carcinoma, Charles G. Cumston, Boston.

8. Surgery of the female bladder and urethra, John B. Murphy, Chicago.

9. Surgery of the ileocecal valve for nonmalignant disease, N. Stone Scott, Cleveland.

10. The curet in postpartum infections of the uterus, D. Tod Gilliam, Columbus.

11. The use of veratrum viride in surgical and obstetrical practice, Chas. L. Bonifield, Cincinnati.

12. Should the uterus and ovaries be removed in cases of double pyosalpinx? C. C. Frederick, Buffalo.

13. Placenta previa, E. T. Abrams, Dollar Bay.

14. The limitations of Cesarean section, E. Gustav Zinke, Cincinnati.

15. Further notes on ovarian grafting, Robt. T. Morris, New York.

16. Conservative surgical treatment of the uterine adnexa, A. P. Clarke, Cambridge.

17. The value of vaginal Cesarean section, M. Stamm, Fremont, O.

18. Hysteria as a result of chronic atrophic parametritis; a contribution to the study of nervous disturbances, W. A. Freund, Berlin.

19. Anesthesia in abdominal surgery, J. J. Gurney Williams, Philadelphia.

20. The technic of gynecological work, A. Vander Veer, Albany.

21. Emergency abdominal surgery at the patient's home—a demonstration, W. G. Macdonald, Albany.

22. Discussion of common causes of death following pelvic and abdominal operations, Joseph Price, Philadelphia.

23. The indications and technic of vaginal drainage for suppuration in the pelvis, A. Goldspohn, Chicago.

24. Infravaginal elongation of the cervix, M. Rosenwasser, Cleveland.

25. Appendicitis, Walter P. Manton, Detroit.

26. Chloroform in labor, Edwin Ricketts, Cincinnati.

27. Study of the symptoms and surgical treatment of intestinal perforation in typhoid fever, W. D. Haggard, Nashville.

28. Symptomatology of the pelvic musculature, Hugo O. Pantzer, Indianapolis.

29. Palliative treatment of cancer of the cervix, Walter B. Chase, Brooklyn.

30. Abdominal versus vaginal hysterectomy in carcinoma where the radical operation is warranted, John B. Deaver, Philadelphia.

31. Hysterectomy in infectious diseases of the uterine appendages, H. C. Deaver, Philadelphia.

32. The scope and limitation of myomectomy in the treatment of fibroid tumors of the uterus, L. S. McMurtry, Louisville.

33. In memoriam,—William E. B. Davis, L. S. McMurtry, Louisville.

34. Penetrating gunshot and stab wounds of the abdomen, with report of cases, John Young Brown, Jr., Saint Louis.

35. Report of abdominal sections during pregnancy, X. O. Werder, Pittsburg.

36. Shortening the round ligaments by the blunt hook method, H. W. Longyear, Detroit.

37. The Gilliam operation; a clinical contribution, Edward J. Ill, Newark.

38. Report of a fourth consecutive successful operation for acute perforated gastric ulcer, with general infection of the peritoneal cavity, Henry Howitt, Guelph, Ont.

39. Coincident tubal and uterine pregnancy, with report of case; remarks, Frank F. Simpson, Pittsburg.

40. Infantile uterus, scanty menstruation, amenorrhea and dysmenorrhea, cured by stem pessaries, J. H. Carstens, Detroit.

During the meeting Dr. J. B. Murphy, by request, will hold a clinic, at which some demonstrations of special interest will be made. Dr. Macdonald will give a demonstration of Emergency Surgery at the Patient's Home, showing operator, assistants, nurses and equipment in full detail, immediately preceding Dr. Murphy's clinic.

All members of the medical profession are cordially invited to attend the scientific sessions.

---

The American Congress on Tuberculosis will hold its next meeting at Washington, April 4, 5 and 6, 1905, under the presidency of Dr. Daniel Lewis, of New York. The president announces Dr. Alfred Meyer, of New York, as chairman of a committee in charge of the section on the sanitarium treatment of tuberculosis.

---

The Mississippi Valley Medical Association will hold its twenty-ninth annual meeting at Memphis, Tenn., October 7, 8 and 9, 1903, under the presidency of Dr. Edwin Walker, of Evansville. The secretary, Dr. Henry E. Tuley, Louisville, should be addressed concerning the program and other details.

The Medical Society of the State of New York will hold an autumn meeting at the New York Academy of Medicine, 17 West 43d street, New York City, Thursday and Friday, October 15 and 16, 1903, under the presidency of Dr. A. T. Bristow, Brooklyn. A cordial invitation is extended to all physicians.

---

The American Electrotherapeutic Association will hold its twelfth annual meeting at the Hotel Kaaterskill, Catskill Mountains, September 2, 3 and 4, 1903, under the presidency of Dr. Daniel Roberts Brower, of Chicago.

---

The Southern Surgical and Gynecological Association will hold its sixteenth annual meeting at Atlanta, Ga., Tuesday, Wednesday and Thursday, December 15, 16 and 17, 1903, under the presidency of Dr. J. Wesley Bovee, of Washington, D. C. The secretary, Dr. William D. Haggard, Nashville, announces that the indications for the approaching meeting are for an unusually full attendance. The headquarters of the association will be at the new Piedmont Hotel.

---

The Medical Association of Central New York will hold its thirty-sixth annual meeting at Auburn, September 22, 1903, under the presidency of Dr. John L. Heffron, of Syracuse. The program is preparing and will be issued early in September.

